# The many-faced KSR1: a tumor suppressor in breast cancer

**DOI:** 10.18632/oncoscience.219

**Published:** 2015-08-25

**Authors:** Hua Zhang, Justin Stebbing, Georgios Giamas

**Affiliations:** Imperial College London, Division of Cancer, Hammersmith Hospital Campus, London, UK

**Keywords:** KSR1, breast cancer, pseudokinase

Emerging evidence supports the dual function of kinase suppressor of Ras 1 (KSR1) as an active kinase and a scaffold, although it has been extensively referred as a pseudokinase, due to the absence of key residues in its catalytic domain [1, 2]. As a scaffolding protein, KSR1 orchestrates the assembly of the protein kinases RAF, mitogen activated protein kinase (MAPK) kinase (MEK), and extracellular signal-regulated kinase (ERK) in the canonical Ras-RAF-MAPKs pathway, in a Ras-dependent manner or upon growth factor treatment [1, 3]. Conversely, structural and biochemical studies reveal that KSR1 is capable of phosphorylating MEK and more importantly, the catalytic activity of KSR is markedly increased when BRAF or inhibitor-bound CRAF is introduced in the complexes [1, 4, 5]. Such findings add complexity to the ERK spatio-temporal pathway control and identify KSR1 as a modulator of these pathways.

In light of its regulatory role in oncogenic Ras-RAF-MAPKs signaling, extensive efforts have attempted to establish KSR1 as an oncogene in Ras-dependent cancers. Indeed, KSR1 has been shown to contribute to oncogenesis in various forms of Ras-activated cancer, including skin, pancreatic and lung carcinomas [1]. First, absence of KSR1 inhibits tumor formation in different Ras-mediated mouse models (KSR1^−/−^), suggesting that KSR1 is required for Ras-transduced MAPK activated tumorigenesis. Moreover, depletion of KSR1 reduces tumor growth of K-Ras-dependent pancreatic and lung cancer xenografts in nude mice, further supporting KSR1 as an oncogene as well as a potential therapeutic target.

However, our own studies reveal an interesting, and yet unexpected role of KSR1 in breast cancer, where Ras mutations are rare. Using tumor tissue microarrays in a large patient cohort with a long term follow-up, we observe that breast cancer patients with high KSR1 had better disease free- and overall survival, results also supported by Oncomine analyses, microarray data and genomic data from paired tumor and cell-free DNA (cfDNA) samples revealing loss of heterozygosity [6]. KSR1 expression is positively associated with breast cancer 1, early onset (BRCA1), BRCA1-associated ring domain 1 (BARD1) and checkpoint kinase 1 (Chk1) levels in breast cancer specimens. Intriguingly, phospho-profiling of major components of the canonical Ras-RAF-MAPKs pathway, including RAF, MEK and ERK, show no significant changes upon KSR1 overexpression or depletion. These results underline its role beyond coordinating MAPKs signaling and challenge its oncogenic function in breast cancer.

Further experiments support a tumor suppressive role of KSR1. Of note, breast cancer cells overexpressing KSR1 form fewer and smaller size colonies compared to the parental ones, while an *in vivo* mouse model also demonstrates that the growth of xenograft tumors overexpressing KSR1 is inhibited. It appears that the tumor inhibitory effect of KSR1 is BRCA1-dependent as shown by *in vitro* 3D-matrigel and soft-agar assays. Consistent with the correlation between KSR1 and BRCA1 in clinical samples, *in vitro* assays demonstrate KSR1 overexpression increases BRCA1 protein levels by decreasing BRCA1 ubiquitination partly through elevating BARD1 protein abundance and the BRCA1-BARD1 interaction. These findings integrate KSR1 into a complicated signaling network involving BRCA1-BARD1 complex. Unquestionably, alternative mechanisms underlying BRCA1's up-regulation by KSR1 other than through BARD1 remain to be examined.

**Figure 1:**
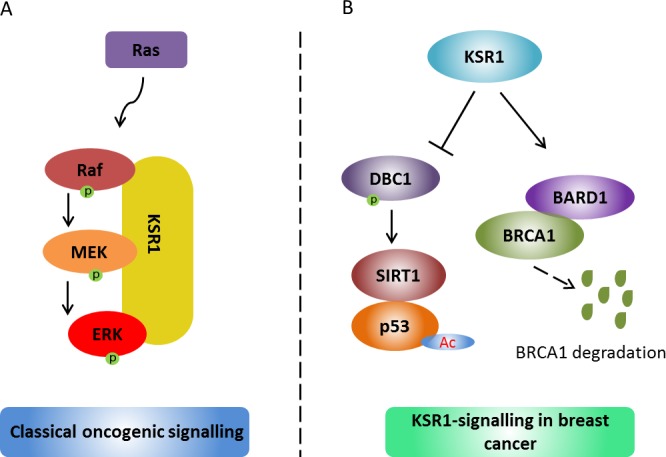
KSR1-regulated signaling in cancer **A.** In Ras-dependent cancers, KSR1 coordinates the classical oncogenic Ras-RAF-MAPKs signaling to promote tumor. **B.** In breast cancer, on one hand, KSR1 represses p53 transcriptional activity through modulation of deleted in breast cancer 1 (DBC1) phosphorylation and the SIRT1-DBC1 complex. More importantly, KSR1 affects BRCA1 expression by decreasing BRCA1 ubiquitination partly through regulating BARD1 protein abundance and the BRCA1-BARD1 interaction

Our current understanding of KSR1's function in breast cancer is far from complete (Figure 1). Puzzlingly, in addition to executing its anti-tumor effects through BRCA1, KSR1 negatively regulates transcriptional activity of p53 by reducing p53 acetylation via modulation of deleted in breast cancer 1 (DBC1) phosphorylation [7]. How can KSR1 up-regulates BRCA1 and inhibits p53 activity at the same time? What determines its preferable binding partners and thus contributes to its inhibitory outcome? An interesting and simple theory might be the following: since DBC1 is a positive regulator of p53 activity and a repressor for BRCA1, it potentially positions KSR1 into a dynamic state connecting p53 and BRCA1. Ultimately, while BRCA1 compensates for the loss of p53 activity, KSR1 tilts the balance scale towards tumor suppression in cancer cells. Further investigation is much needed to address the underlying mechanisms. In addition, evidence regarding other aspects of its action are evolving: a very recent study characterizing the proteomic profile of KSR1-regulated signaling in response to genotoxic agents in breast cancer illustrates a broad functional network conferred by KSR1, highlighting its importance in the chemotherapy response [8]..

A better understanding of the molecular determinants of the distinctive behaviour of KSR1 in different types of cancer is essential to the optimal targeting of this dual functional kinase-scaffold protein. Our studies suggest the tumor suppressive action of KSR1 and its clinical relevance in patient stratification, placing KSR1 in the major oncoprotein pathways.
